# Using the spike protein feature to predict infection risk and monitor the evolutionary dynamic of coronavirus

**DOI:** 10.1186/s40249-020-00649-8

**Published:** 2020-03-25

**Authors:** Xiao-Li Qiang, Peng Xu, Gang Fang, Wen-Bin Liu, Zheng Kou

**Affiliations:** Institute of Computing Science and Technology, Guangzhou University, Guangzhou, 510006 China

**Keywords:** Coronavirus, Cross-species infection, Spike protein, Machine learning

## Abstract

**Background:**

Coronavirus can cross the species barrier and infect humans with a severe respiratory syndrome. SARS-CoV-2 with potential origin of bat is still circulating in China. In this study, a prediction model is proposed to evaluate the infection risk of non-human-origin coronavirus for early warning.

**Methods:**

The spike protein sequences of 2666 coronaviruses were collected from 2019 Novel Coronavirus Resource (2019nCoVR) Database of China National Genomics Data Center on Jan 29, 2020. A total of 507 human-origin viruses were regarded as positive samples, whereas 2159 non-human-origin viruses were regarded as negative. To capture the key information of the spike protein, three feature encoding algorithms (amino acid composition, AAC; parallel correlation-based pseudo-amino-acid composition, PC-PseAAC and G-gap dipeptide composition, GGAP) were used to train 41 random forest models. The optimal feature with the best performance was identified by the multidimensional scaling method, which was used to explore the pattern of human coronavirus.

**Results:**

The 10-fold cross-validation results showed that well performance was achieved with the use of the GGAP (g = 3) feature. The predictive model achieved the maximum ACC of 98.18% coupled with the Matthews correlation coefficient (MCC) of 0.9638. Seven clusters for human coronaviruses (229E, NL63, OC43, HKU1, MERS-CoV, SARS-CoV, and SARS-CoV-2) were found. The cluster for SARS-CoV-2 was very close to that for SARS-CoV, which suggests that both of viruses have the same human receptor (angiotensin converting enzyme II). The big gap in the distance curve suggests that the origin of SARS-CoV-2 is not clear and further surveillance in the field should be made continuously. The smooth distance curve for SARS-CoV suggests that its close relatives still exist in nature and public health is challenged as usual.

**Conclusions:**

The optimal feature (GGAP, g = 3) performed well in terms of predicting infection risk and could be used to explore the evolutionary dynamic in a simple, fast and large-scale manner. The study may be beneficial for the surveillance of the genome mutation of coronavirus in the field.

## Background

Coronavirus (CoV) belongs to the order Nidovirales and can infect humans, mammals, and birds [1]. The viral genome is composed of a positive stranded RNA, and its structures vary. The family Coronavirinae is divided into four genera: α, β, γ, and δ [2]. There are seven human coronaviruses: 229E (α-CoV), NL63 (α-CoV), OC43 (β-CoV), HKU1 (β-CoV), MERS-CoV (β-CoV), SARS-CoV (β-CoV), and SARS-CoV-2 (β-CoV). MERS-CoV, SARS-CoV and SARS-CoV-2 can infect humans and induce serious pneumonia with many fatal cases [3]. SARS-CoVs induced an epidemic in the world, and 774 fatal cases were reported [3]. Now, SARS-CoV-2 is still circulating in China [4–6].

As considerable coronaviruses have been isolated from bats and other animals, it is believed that there is a viral gene reservoir in wild animals [7]. Coronavirus can directly cross the species barrier and infect humans with high fatality [8]. As the antigen is novel for a human host, public health is being seriously challenged. The infection risk of coronavirus in animals should be analyzed and a prediction model should be constructed for early warning. For this purpose, machine-learning methods appear to be ideal tools [9, 10]. The spike protein on the surface of the viral particle plays key roles in the binding of the cell receptor and membrane fusion [3, 11], by which the host range is firmly determined [8]. In this study, we screened the features of the spike protein using three encoding algorithms and predicted the cross-species infection of coronaviruses with the random forest method. Moreover, the optimal feature (G-gap dipeptide composition, GGAP, g = 3) was used to explore the dynamic of evolution in a simple, fast and massive manner.

## Methods

### Dataset

The protein sequences of 2666 coronaviruses were collected from 2019 Novel Coronavirus Resource (2019nCoVR) Database of China National Genomics Data Center (NGDC, https://bigd.big.ac.cn/ncov) on Jan 29, 2020 [12]. These strains had full length genomes and were isolated between 1941 and 2020, and included SARS-CoV-2 strains. The information related to these strains was summarized in Additional file 1. The 507 human-origin coronaviruses were regarded as positive samples, whereas the 2159 non-human-origin coronaviruses were regarded as negative.

### Feature encoding algorithms

To capture the key information of the spike protein, we used three encoding algorithms from multiple perspectives, that is compositional information, position-related information and physicochemical properties (Table 1). The optimal feature with the best performance was shown by the multidimensional scaling method in R (MDS, https://cran.r-project.org/web/packages/MASS/index.html). The details of the feature encoding algorithms used to encode the spike protein into feature vectors are listed below.

**Table 1:** Summary of feature descriptors

Feature	Type	Dimension	Feature	Type	Dimension
1	PseAAC (λ = 1)	21	22	GGAP (g = 0)	400
2	PseAAC (λ = 2)	22	23	GGAP (g = 1)	400
3	PseAAC (λ = 3)	23	24	GGAP (g = 2)	400
4	PseAAC (λ = 4)	24	25	GGAP (g = 3)	400
5	PseAAC (λ = 5)	25	26	GGAP (g = 4)	400
6	PseAAC (λ = 6)	26	27	GGAP (g = 5)	400
7	PseAAC (λ = 7)	27	28	GGAP (g = 6)	400
8	PseAAC (λ = 8)	28	29	GGAP (g = 7)	400
9	PseAAC (λ = 9)	29	30	GGAP (g = 8)	400
10	PseAAC (λ = 10)	30	31	GGAP (g = 9)	400
11	PseAAC (λ = 11)	31	32	GGAP (g = 10)	400
12	PseAAC (λ = 12)	32	33	GGAP (g = 11)	400
13	PseAAC (λ = 13)	33	34	GGAP (g = 12)	400
14	PseAAC (λ = 14)	34	35	GGAP (g = 13)	400
15	PseAAC (λ = 15)	35	36	GGAP (g = 14)	400
16	PseAAC (λ = 16)	36	37	GGAP (g = 15)	400
17	PseAAC (λ = 17)	37	38	GGAP (g = 16)	400
18	PseAAC (λ = 18)	38	39	GGAP (g = 17)	400
19	PseAAC (λ = 19)	39	40	GGAP (g = 18)	400
20	PseAAC (λ = 20)	40	41	GGAP (g = 19)	400
21	AAC	20			

*GGAP* G-gap dipeptide composition, *PseAAC* Pseudo-amino-acid composition, *AAC* Amino acid composition

#### Amino acid composition

Amino acid composition (AAC) is a simple but commonly used feature descriptor for sequence analysis and model construction. For a total of 20 amino acid types, the AAC descriptor calculates the frequency of each type of amino acid. For example, if the amino acid type i occurs n_i_ times in the protein sequence, then the frequency of i is denoted by f(i) = n_i_/L, where L is the protein length. For a given strain, we yielded a 20-dimensional feature vector by computing the frequencies of 20 different amino acids.

#### Parallel correlation-based pseudo-amino-acid composition

Parallel correlation-based pseudo-amino-acid composition (PC-PseAAC) measures the parallel correlation between any two amino acids in a protein sequence [13]. For a given strain P, the PC-PseAAC feature vector is represented by

$$ PC- PseAAC={\left[{fv}_1,\dots, {fv}_{20},{fv}_{20+1},\dots, {fv}_{21+\uplambda}\right]}^T $$

where

$$ {fv}_u=\left\{\begin{array}{c}\frac{f_u}{\sum_{i=1}^{20}{f}_i+w{\sum}_{j=1}^{\uplambda}{\theta}_j},1\le u\le 20\\ {}\frac{w{\theta}_{u-20}}{\sum_{i=1}^{20}{f}_i+w{\sum}_{j=1}^{\uplambda}{\theta}_j},20+1\le u\le 20+\uplambda \end{array}\right. $$

where u is an integer; fv_u_ (1 ≤ u ≤ 20) represents the normalized appearance frequency of the 20 amino acids in the spike protein of P; λ represents the highest tier of the correlation along P; and θj (j = 1, 2, ..., λ) is the correlation function that measures the j-tier sequence-order correlation between all the j-th most contiguous residues along P. θj is calculated using the following formula:

$$ {\theta}_j=\frac{1}{L}\ \sum \limits_{i=1}^L\frac{1}{5}\sum \limits_{m=1}^5{\left[{H}_m\left({P}_{i+j}\right)-{H}_m\left({P}_i\right)\right]}^2 $$

where Hm (Pi) (m = 1,2,3,4,5) represents the polarity, secondary structure, molecular volume, codon diversity, and electrostatic charge corresponding to the i-th amino acid Pi in the protein sequence P, respectively [14]. If I + j > L, then I + j equals I + j - L.

#### G-gap dipeptide composition

The G-gap dipeptide composition (GGAP) achieves the dipeptide composition coupled with local order information of any two interval residues within the spike sequence. It is formulated as

$$ GGAP(g)=\left({fv}_1^g,{fv}_2^g,\dots, {fv}_{400}^g\right) $$

where ${fv}_i^g$ is the occurrence frequency of the i-th (i = 1,2, ...,400) G-gap dipeptide, which is computed as

$$ {fv}_i^g=\frac{O_i^g}{\sum_{i=1}^{400}{O}_i^g} $$

where ${O}_i^g$ represents the occurrence number of the i-th G-gap dipeptide in the spike protein. The dimension of the GGAP feature vector is 20 × 20 = 400.

### Machine learning

**Fig. 1:**
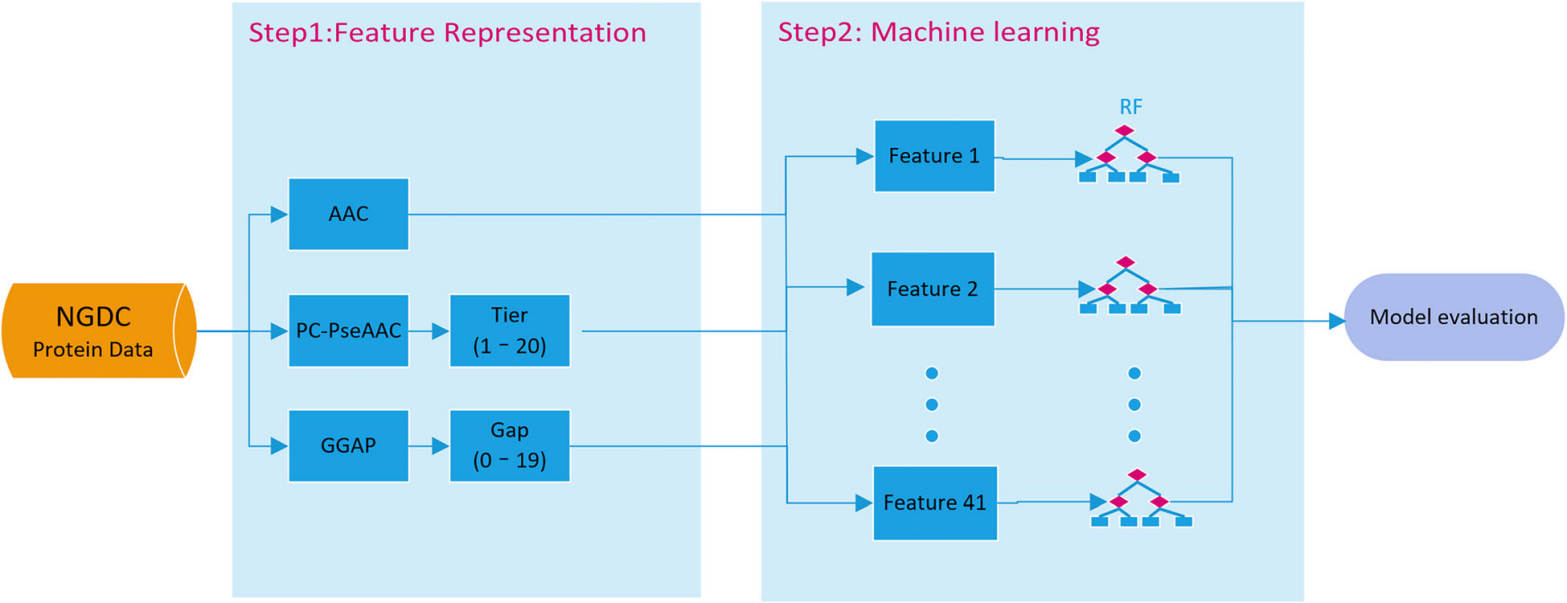
Schematic framework of machine learning. First, feature representations from three feature descriptors are obtained. Second, the RF method is used to train and test the dataset and make predictions for cross-species transmission of coronavirus. NGDC: National Genomics Data Center; AAC: Amino acid composition; PC-PseAAC: Parallel correlation-based pseudo-amino-acid composition; GGAP: G-gap dipeptide composition; RF: Random forest

The framework for the overall prediction is shown in Fig. 1. Two main steps are included: feature representation and machine learning. First, feature representations from three feature descriptors are achieved using the algorithm as described above. Second, the random forest (RF) method is used to train and test the prediction models.

As robust and well performance in the field of machine learning, the RF has been widely used to model biological data. In this study, the RF algorithm is used to construct models and make predictions for the cross-species transmission of coronavirus. The RF behaves like an ensemble algorithm and proposes a set of decision trees, which are grown by a subset of features. The RF repeats the computing process many times and then makes a final prediction on each sample. The final prediction can simply be the mean of each prediction with bootstrapping algorithm. In this study, the RF algorithm in the R environment was used [15]. All the experiments in the study were conducted under R 3.5.0 with default parameters (tree number = 500). To reduce the bias of unbalanced sample number, the positive samples were increased fourfold by the direct duplication of their protein sequences. The 10-fold cross validation method was used to evaluate the predictive performance. Platt scaling was used to transform the output of the RF model into a probability over two classes and evaluated the infection risk of coronaviruses.

### Performance evaluation metrics

Four commonly used metrics for model performance evaluation, that is, sensitivity (SN), specificity (SP), accuracy (ACC) and Matthews correlation coefficient (MCC), were used in the study. The details are listed as follows:

$$ \left\{\begin{array}{c} SN=\frac{TP}{TP+ FN}\times 100\%\\ {} SP=\frac{TN}{TN+ FP}\times 100\%\\ {} ACC=\frac{TP+ TN}{TP+ TN+ FP+ FN}\times 100\%\\ {} MCC=\frac{TP\times TN+ FP\times FN}{\sqrt{\left( TP+ FN\right)\ \left( TP+ FP\right)\ \left( TN+ FN\right)\ \left( TN+ FP\right)}}\end{array}\right. $$

where TP indicates true positive, which is the number of correctly predicted true strains with the phenotype of cross-species transmission; TN represents true negative, which is the number of correctly predicted true strains without the phenotype of cross-species transmission; FP represents false positive, which is the number of strains without the phenotype of cross-species transmission predicted to be strains with the phenotype of cross-species transmission; and FN represents false negative, which is the number of strains with the phenotype of cross-species transmission predicted to be strains without the phenotype of cross-species transmission. The SE and SP metrics measure the predictive ability of the model for positive and negative cases, respectively. The other two measures, ACC and MCC, are used to evaluate the overall performance of the model. Regarding all the metrics above, the higher their scores, the better performance of the model have.

In this study, we also used the receiver operating characteristic curve (ROC) to evaluate the overall performance of a binary classifier system [16]. It is generated by plotting the true positive rate (TPR) against the false positive rate (FPR) under different classification thresholds. TPR is also known as sensitivity, as described in the above section, whereas FPR can be calculated as specificity.

## Results

### Screening of the optimal feature

**Fig. 2:**
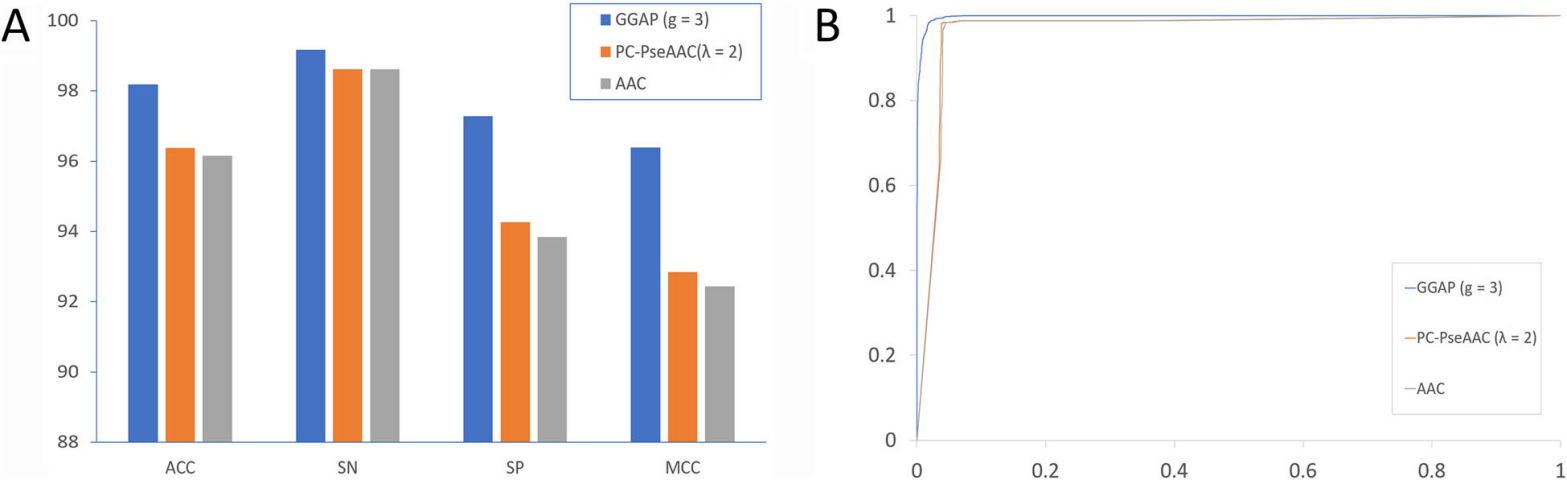
Predictive performance of feature representations. **a** Ten-fold cross-validation results. **b** Receiver operating characteristic curves generated by plotting the true positive rate (TPR) against the false positive rate (FPR) under different classification thresholds. ACC: Accuracy; SN: Sensitivity; SP: Specificity; MCC: Matthews correlation coefficient; AAC: Amino acid composition; GGAP: G-gap dipeptide composition; PC-PseAAC: Parallel correlation-based pseudo-amino-acid composition

As described in the section Feature encoding algorithms, we used three feature encoding algorithms from multiple perspectives, that is, compositional information and position-related information, in addition to physicochemical properties. A total of 41 features were used to train the prediction models as shown in Table 1. The performances of the protein features were different and the prediction results for the features with the best performance for each type are shown in Table 2. As shown in Table 2 and Fig. 2a, the predictive model achieved the maximum ACC of 98.18% coupled with the MCC of 0.9638 when the feature GGAP (g = 3) was selected. The performance varied from 96.15 to 98.18% for ACC and from 0.9243 to 0.9638 for MCC. This indicated that the feature GGAP with parameter 3 had the optimal representation ability to distinguish coronaviruses with different phenotypes of cross-species transmission. For the receiver ROC shown in Fig. 2b, the feature GGAP (g = 3) also performed better than the other features (PC-PseAAC or AAC). The optimal GGAP feature representation could be explored to monitor the evolutionary dynamics of coronavirus.

**Table 2:** Results of feature representations

Feature	ACC	SN	SP	MCC	TP	TN	FP	FN
GGAP (g = 3)	98.18	99.16	97.26	0.9638	2011	2100	59	17
PC-PseAAC (λ = 2)	96.36	98.61	94.25	0.9284	2000	2035	124	28
AAC	96.15	98.61	93.83	0.9243	2000	2026	133	28

*ACC* Accuracy, *SN* Sensitivity, *SP* Specificity, *MCC* Matthews correlation coefficient, *TP* True positive, *TN* True negative, *FP* False positive, *FN* False negative, *GGAP* G-gap dipeptide composition, *PC-PseAAC* Parallel correlation-based pseudo-amino-acid composition, *AAC* Amino acid composition

### Patterns of human coronavirus

**Fig. 3:**
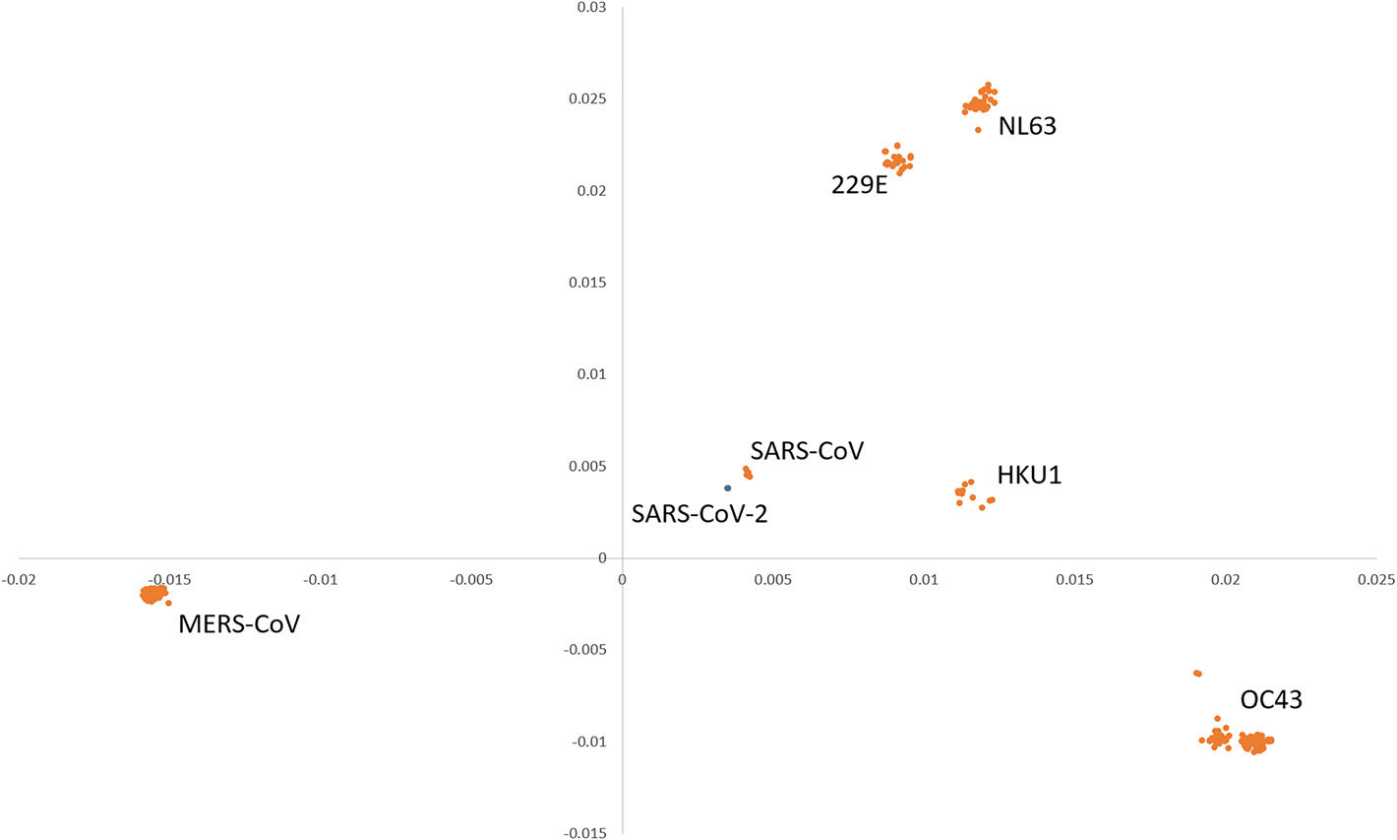
Patterns of human coronavirus clustered using the multidimensional scaling method. The x and y coordinates denote the first main factor and second main factor, respectively. SARS-CoV-2 is indicated by the blue solid circle

As shown in Table 2 and Fig. 2, the GGAP (g = 3) had the best performance and is proposed to monitor the evolutionary dynamics of coronavirus. The features of the 507 human samples in our dataset were used to show the patterns with the multidimensional scaling method. Seven clusters for 229E (α-CoV), NL63 (α-CoV), OC43 (β-CoV), HKU1 (β-CoV), MERS-CoV (β-CoV), SARS-CoV (β-CoV), and SARS-CoV-2 (β-CoV) were formed obviously (Fig. 3). The clusters for 229E and NL63 were closed and located in the upper right of the figure. The cluster for SARS-CoV-2 was very close to that for SARS-CoV, which suggests that both viruses have the same human receptor (angiotensin converting enzyme II, ACE2). The two clusters for MERS and OC43 were far away from SARS-CoV and SARS-CoV-2.

### Evolutionary dynamics of SARS-CoV and SARS-CoV-2

**Fig. 4:**
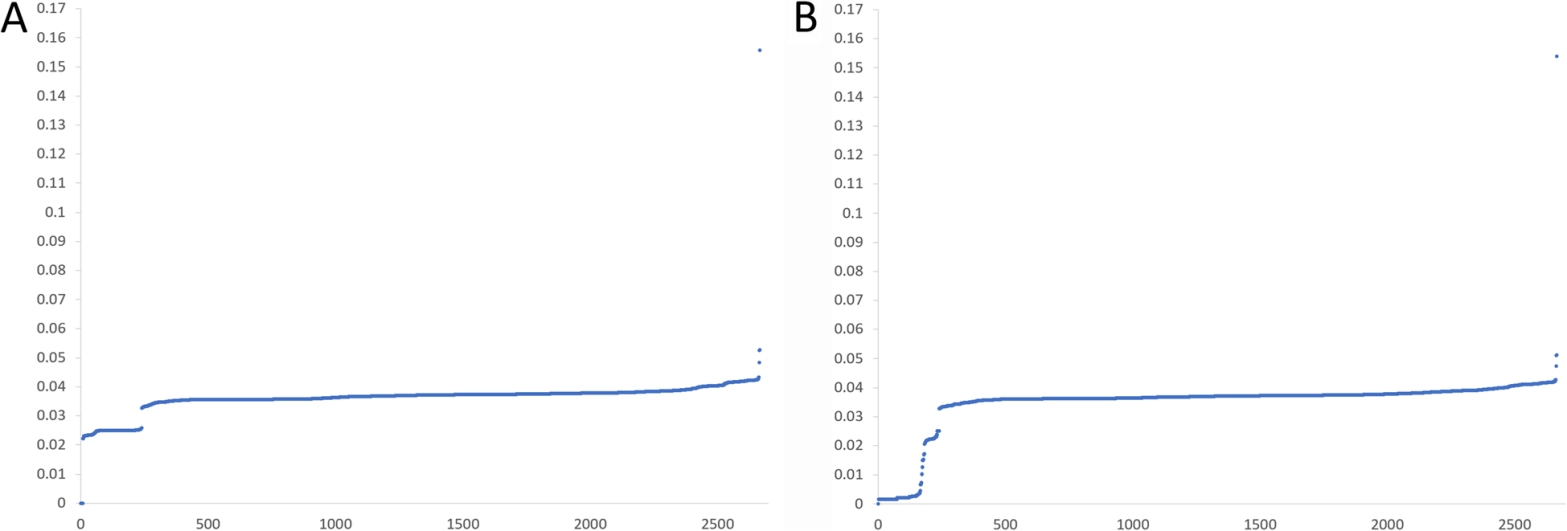
Evolutionary dynamic of SARS-CoV-2 and SARS-CoV. **a** Euclidean distance between SARS-CoV-2 and other coronaviruses in the dataset. **b** Euclidean distance between SARS-CoV and other coronaviruses in the dataset. The x and y coordinates denote the strain number and Euclidean distance based on the GGAP (g = 3) feature, respectively

The optimal GGAP feature performed well in terms of predicting infection risk and was used to explore the dynamic of evolution in a simple, fast and massive manner. Based on the GGAP (g = 3) feature, we computed the Euclidean distance of SARS-CoV-2 and SARS-CoV from other coronaviruses in the dataset to explore the evolution dynamic, separately. As shown in Fig. 4a, the distance curve between SARS-CoV-2 and other coronaviruses had two gaps. The ‘big’ gap with values from 0 to 0.02 suggests that the SARS-CoV-2 have no close relation with other isolated coronaviruses. As shown in Fig. 4b, the distance curve between SARS-CoV and other coronaviruses also had a gap of value 0.03, which is similar to that of SARS-CoV-2. The two gaps at 0.03 suggest that coronaviruses close to SARS-CoV-2 s or SARS-CoVs form a separate group. We further checked the coronaviruses close to SARS-CoV-2 and SARS-CoV (< 0.03) and found that these close relatives were the same. The results were similar to those from the MDS method and confirmed that SARS-CoV-2 s and SARS-CoVs have the same origin. Moreover, the big gap at 0.02 suggests that the origin of SARS-CoV-2 s is not clear and further surveillance in the field should be made continuously. The smooth curve for SARS-CoVs shows that its close relatives still exist in nature and public health is challenged as usual.

### Implementation of the prediction tool

We used the Python language to establish an easy-to-use tool that implements our predictor, which is freely accessible via https://github.com/kouzheng/CovPred-FL and can run in a simple, fast and massive manner. For the convenience of researchers, we provide guidelines on how to use the tool to obtain the desired results: (1) Users need to prepare the query sequences in the FASTA format. Examples of FASTA formatted sequences can be found in the directory mentioned previously. (2) Users need to input the name of the query file and set the confidence parameter before running predictions. The prediction confidence has a range from 0.0 to 0.5. The lower the confidence set by users, the more sensitive the predictions obtained by users. The predicted label for ‘H’ means the phenotype of cross-species transmission while label for ‘N’ means not. The probability for infection risk is also listed in the result file. The file for the features of the query sequence is created to facilitate further analysis.

## Discussion

At present, SARS-CoV-2 is still circulating in China and the epidemic causes widespread social concern in the world [17, 18]. As considerable coronaviruses have been isolated from bats and other animals, it is believed that there is a viral gene reservoir in wild animals [7]. Coronavirus can directly cross the species barrier and infect humans with a severe syndrome [8]. As an antigen that is novel for a human host, public health is being challenged seriously. With the use of the viral spike protein, in this study, the infection risk of non-human-origin coronavirus was analyzed and a prediction model was constructed for early warning to prevent disease.

The spike protein on the surface of the viral particle plays key roles in the binding of the cell receptor and membrane fusion [3, 11], by which the host range is firmly determined [8]. In the study, we choose the spike protein as a candidate target to predict the cross-species infection of coronaviruses using the RF method. For the spike protein of coronavirus, the sequence lengths were different and sequence identities were very low between remote relatives, which caused the problem of alignment and challenged the algorithms used to model biology data. For analysis and modeling in a simple, fast and massive manner, we used three different feature encoding algorithms from multiple perspectives, such as compositional information and, position-related information, in addition to physicochemical properties. The computation of protein features did not require multiple sequence alignment and reduced the computational complexity.

A total of 41 features were used to train the prediction models. The best predictive model achieved the maximum ACC of 98.18% coupled with the MCC of 0.9638 when the feature GGAP (g = 3) was selected, which indicated that the feature GGAP with parameter 3 had the optimal representation ability to distinguish coronaviruses with different phenotypes of cross-species transmission. As shown in Table 2, the number of false positives was 59. The reason for the false positives may be the sporadic infection of coronavirus that originated from an animal or a conflicting description of the ability of human receptor binding. With the improvement of annotation in the database, the false rate could be reduced [19].

The MDS results were similar to those from traditional evolution analysis [1, 3, 5], which confirmed that the screening of the GGAP (g = 3) feature was reasonable for the prediction of cross-species transmission. Moreover, we computed the Euclidean distance of SARS-CoV-2 and SARS-CoV from other coronaviruses in the dataset to explore the evolution dynamic. The big gap of 0.02 suggests that the origin of SARS-CoV-2 is not clear and further surveillance in the field should be made continuously. As considerable work on molecular epidemiology in the field has been conducted recently, more than 2000 genome sequences of coronavirus isolated from animals have been identified. In addition to various bat species, other animals should be suspected as direct hosts for SARS-CoV-2. According to the smooth curve for SARS-CoVs, the fact should be noted that its close relatives still exist in nature and public health is challenged as usual.

Although many proteins contribute to the procedure of virus production and host invasion, the spike protein is the most important factor to determine host range [8, 19, 20]. A long sequence of the viral genome should be considered in further study to increase the performance of the prediction model. However, applying the algorithm for about 30 000 dimensions of data and small number of samples will be a challenge. In the study, the infection risk of non-human-origin coronavirus was evaluated for early warning and good performance was achieved. The main limitation was that only viral spike proteins were used to build the prediction model and social factors, such as traffic conditions, population size, and citizens habits in daily life, were not involved. Although high risk could be predicted in the view of the pathogen, comprehensive judgment should be used to prevent disease in the future.

## Conclusions

In this paper, we presented a predictor for the identification of the transmission phenotype of coronavirus. The major contribution of this predictor is that a set of informative features of viral proteins from 41 feature descriptors, such as compositional, position-specific and physicochemical information, were learned using a machine learning algorithm. The 10-fold cross-validation results showed that good performance was achieved with the use of the GGAP (g = 3) feature. The optimal feature performed well in terms of predicting infection risk and was used to explore the dynamic of evolution in a simple, fast, and massive manner. This study may be beneficial for coronavirus surveillance and future study on the cross-species transmission of coronavirus.

## Supplementary information

**Additional file 1.**

**Additional file 2.**

## Data Availability

The protein sequences of 2666 coronaviruses analyzed during the current study are available in the NGDC’s 2019nCoVR Database, https://bigd.big.ac.cn/ncov [12]. The nomenclature for coronavirus in the dataset is provided as Additional file 1. The clustering details for the MDS method is provided as Additional file 2.

## Abbreviations

AAC: Amino acid composition
ACC: Accuracy
CoV: Coronavirus
FPR: False positive rate
GGAP: G-gap dipeptide composition
MCC: Matthews correlation coefficient
MDS: Multidimensional scaling
MERS-CoV: Middle East respiratory syndrome coronavirus
NGDC: National Genomics Data Center
PC-PseAAC: Parallel correlation-based pseudo-amino-acid composition
RF: Random forest
ROC: Receiver operating characteristic
SARS-CoV: Severe acute respiratory syndrome coronavirus
SARS-CoV-2: Severe acute respiratory syndrome coronavirus 2
SN: Sensitivity
SP: Specificity
TPR: True positive rate
